# Improving dose calculations on tomotherapy MVCT images

**DOI:** 10.1120/jacmp.v13i6.3986

**Published:** 2012-09-06

**Authors:** F. Crop, A. Bernard, N. Reynaert

**Affiliations:** ^1^ Department of medical physics Université Lille 1 Lille France

**Keywords:** MVCT, calibration problem, dose calculations, tomotherapy

## Abstract

The purpose of this investigation was the creation of a new protocol allowing more precise dose calculations on megavoltage CT (MVCT) images for tomotherapy, both for adaptive and StatRT planning. Daily MVCT images offer, next to positioning purposes, the possibility for daily dose check and adaptive planning. Dose calculations use the image value to density table (IVDT) to calculate physical densities from Hounsfield Units (HUs). These measured HUs change over time, leading to a dose calculation error. We noticed dose calculation variations due to IVDT changes of: 0.2% dose during a day, up to 1.6% dose from long‐term variations, and up to 1.5% dose due to technical interventions. An analysis was performed applying the general methodology of a calibration problem. A model $\text{HU}=b\rho^{c}‐1020$ was obtained using a weighted least squares inverse prediction method (HU as function of density) taking into account the heteroscedasticity. The *b* parameter is the major variable and depends also on the dose rate (DR). We demonstrate the correction for DR variations and the constance of the *c* parameter. Instead of scanning the whole tissue characterization phantom daily, we propose a simplified daily protocol: (a) morning airscan‐like procedure with only two inserts on the table (defining b and thus the IVDT curve), (b) DR variations throughout the day can be corrected for using the DR model. A patient‐specific protocol for which two inserts next to the patient are scanned could also be used, but results in equal uncertainties and is less practical. Therefore we recommend the morning procedure with dose rate variation correction. Applying the proposed transformations and the model, the correct IVDT of the moment can be reconstructed, with a simple measurement in the morning, and corrected with DR changes during the day. This corresponds with a linear mapping in time of the proposed IVDT function. The dosimetric variation is hereby reduced from up to 3% to 0.4 % for the tested pelvic and head‐and‐neck cases. In practice, several IVDT curves corresponding to “b” values can be entered. The correct IVDT curve of that moment can then be chosen from the list. Instead of the two high‐density inserts *on table*, any calibrated single density phantom could be used in order to create the IVDT curve of the day, but it should have a larger size than the current inserts.

PACS number: 87.55.Gh

## I. INTRODUCTION

The TomoTherapy system^(1)^ uses megavoltage computed tomography (MVCT) images for positioning.^(2)^ These images can also be used for dose calculations, adaptive planning, and StatRT (direct) planning.^(3)^ An image value to density table (IVDT) converts the image Hounsfield Units (HU) into mass densities.^4^, ^5^, ^6^ These densities are then used for dose calculations. However, there is an important variation in time for the HU values and thus the IVDT.^7^, ^8^, ^9^ If the recalculated densities are incorrect, a dosimetric uncertainty is induced. The dose differences seen for the recalculations are thus not only related to patient changes. Up to 3% dose differences were observed due to dose rate instabilities^(7)^ and important changes were observed after a target change.^(9)^ Incorrect decisions (both false positives and negatives) regarding replanning a patient can be associated to this uncertainty on the IVDT and resulting dose calculations.

The IVDT curve can be entered manually into the TomoTherapy system by measuring HUs of inserts with different densities inside the cheese tissue characterization phantom. The curve is then created point‐by‐point, with linear interpolation between consecutive points.

Statistically, this is not the most optimal method, especially when the uncertainty on every point is different (heteroscedasticity). The system calculates densities from the HUs, but the uncertainties are linked to the HUs. Basically, this comes down to the famous ‘calibration problem’ in which the problem is first inversed: a model is created to calculate HUs from densities using weighted least squares (WLS) linear regression (inverse prediction using uncertainty per data point). This model is then inversed to obtain a practical model.^(10)^

We investigate two possible protocols to improve calculations on MVCT images:

1 Preparation (once for both):
  a) Measure HU values *in phantom* and calculate the b and c parameter of the response curve $HU=b*\rho^{c}‐1020$ for the machine. Keep the c parameter (exponent) of this full IVDT calibration.
  b) Measure HU values *on table* with a scan after the phantom scan. Calculate HU difference of inserts 1.56 and 1.823 *on table – in phantom*.
  c) Measure the dose rate dependence of the IVDT table by changing the PfN values
2. Daily protocol:
  (a) After the daily airscan, the two high‐density inserts are also scanned on the table (or small support).
  (b) Fit the b parameter of the model to the adjusted HU values *(on table – in phantom)* of the inserts ($\text{HU}=b\rho^{c}‐1020$).
  (c) If the dose rate changes during the day, apply the dose rate transformation
2. Patient specific:
  (a) When scanning patients, put the two inserts next to the patient (or further in the gantry).
  (b) Recalculate the *in phantom* value. Fit the b parameter of the model to the adjusted HU values *(on table – in phantom)* of the inserts ($\text{HU}=b\rho^{c}‐1020$). The correct density curve is then obtained as $\rho {=}^{c}\sqrt{\frac{HU+1020}{b}}$

For this investigation, several measurements were performed to quantify the uncertainties and to generate a model for the density‐HU curve and dose rate transformation.

Normal linear regression can be performed, in order to obtain c and/or b, on the function ln(HU+1020)=c*ln(ρ)+ln(b).

## II. MATERIALS AND METHODS

Measurements were performed on a Hi⋅Art TomoTherapy machine (Accuray, Sunnyvale, California), running version 4.0.3 with a Siemens MVCT detector style 2 (J04 setting) (Siemens Medical Solutions, Malvern, PA). The Tomotherapy Cheese tissue characterization phantom (Gammex RMI, Middelton, WI) was used with inserts with mass densities between 0.29 and $1.823\;g/{\text{cm}}^{3}$. Distilled water was used as reference instead of the 1.017 solid water density rod. As we had two sets of inserts available, we made a mix with certain inserts doubled (20 total).

Some of the inserts were listed with a slightly different density, others came with the same listed density. The inserts were also used in a setup on the table, without surrounding phantom.

Attenuation coefficients in MVCT imaging are mainly dictated by the same interactions as the radiation beam: the presence of heavier atoms (Ca) in density rods exhibits almost no influence, contrary to the behavior for kVCT scans^(11)^ (photo electric interactions). The rods with densities between 0.9 and 1.1 were used as we saw (1) no large variances; (2) these variances are less important in our proposed response curve (which is a drawback for linear interpolation); and (3) these values showed no significant deviations from the behavior of the other inserts or water. For kVCT scans, there are more important differences due to the composition of the inserts; therefore, TomoTherapy recommends not to use rods with HU between ± 100 HUs for kVCT.

Normal slice thickness was used (multiple linear regression using dummy variables indicated no statistical difference between slice thicknesses).

Statistical analysis was performed in “The R project”.^(12)^ Image analysis was performed with OsiriX (The OsiriX Foundation, Geneva, Switzerland) and Amide. Fixed cylindrical geometries $\left(\pm \;5\;{\text{cm}}^{3}\right)$ within the inserts, omitting the borders, were used to obtain the average HU values.

The following measurements and analysis were performed:

- Creation of the response curve
  1. Determination of the heteroscedasticity of the data (variable variance per insert)
  2. Fit of model to the HU‐ρ relation: WLS fitting
- Stability:
  1. Short term: consecutive scans (20 scans times 3)
  2. Daily term: morning‐evening and before‐after airscan (evening)
  3. Long term: several months + interventions
- Transformation of inserts *on table* to *in phantom*
- Dose rate dependence of the IVDT curve
- Verification of the proposed protocols:
  1. Base verification kV‐MV dose calculations
  2. Application of proposed protocols on head‐and‐neck phantom, and patient cases (6 H&N and 6 pelvic region cases):
    - Morning protocol: scan of 2 inserts on the table
    - Patient specific protocol: using 2 inserts next (or above) the patient

## A. Creation of the response curve

### A.1 Heteroscedasticity of measured HU values

The heteroscedasticity is the variable variance of the insert's HU values, depending on density. It was evaluated from one of the 20 successive MVCT sets. A bootstrap method was applied for the estimation of the weight factors for every insert: for every density/insert, the 20‐point dataset was resampled into a new dataset. We then used this set to estimate the variance on the variance. These variances are not normally distributed. Therefore, a bias‐corrected, accelerated 95% confidence interval was applied.^13^, ^14^

### A.2 General Model

The snapshot model was created using one set of 20 successive measurements (for the runs test, see Material and Methods Section B.1). A weighted least squares fit was applied to the HU‐ρ relationship, using the heteroscedasticity from previous paragraph — points with a high uncertainty get lower weight for fitting purposes. We tested several models: polynomial and power functions.

A verification of the slice thickness was performed, using multiple linear regression (MLR) with dummy variables. MLR with dummy variables tests if a categorical variable (like slice thickness or before‐after airscan) plays a role in the IVDT curve. The MLR method using dummy variables adds an additional variable for each thickness (example of coding: Coarse: 0 0, Normal: 0 1, Fine 1 0). This variable is taken as a regression variable. If the coefficient of this variable is statistically significant (95% level), the $H_{0}$ hypothesis of equality is rejected, and the slice thickness is evaluated as resulting in a different density–HU curve.

### B. IVDT stability

The IVDT changes were first investigated on short term, daily and long term. Afterwards, several real patient cases were recalculated using the different IVDT curves and compared (same MVCT with different IVDT curves) in order to assess the induced dosimetric uncertainties in reality (daily, long term, technical intervention: see patient evaluation). The most extreme cases were taken.

#### B.1 Short term: Wald‐Wolfowitz runs test

The importance of this test was to validate that on short term, there were no drifts or autocorrelations. A runs test evaluates if the deviation from the mean of each series of measurements of an insert corresponds to a random distribution around the mean of every insert. A run corresponds to a series of HU values above (or below) the mean of the insert. A series of 20 successive MVCT scans, immediately following each other, was performed. For every insert, the deviation from the mean is calculated and a runs test is applied. This test was performed three times on different days.

The sample test statistic is:

(1) $$Z=\frac{X-E(X)}{\sqrt{\sigma {\left(X\right)}^{2}}}$$

The expected number of runs (E(X)) and its variance (σ^2^(X)) is calculated, using the number of positive (P) and negative runs (N):

(2) $$E(X)=\frac{2PN}{P+N}+1$$

(3) $$\sigma^{2}(X)=\frac{2PN(2PN-(P+N)}{{\left(P+N\right)}^{2}(P+N-1)}$$

#### B.2 Daily variation, before/after airscan: MLR with dummy variables

Two series of three measurements were performed, during the morning and during the evening after eight hours of treatments. This was performed twice, on different days. Two series of ten measurements were performed during the evening, one before a new airscan, one after a new airscan. The analysis was performed using MLR with dummy variables (see Material and Methods Section A.2).

#### B.3 Long‐term stability and stability after interventions

The IVDT curve can change significantly, leading to dose differences of several percentages.^7^, ^9^ IVDT curves were measured during several months. During this period, some major magnetron instabilities were present. A new magnetron was installed leading to better beam stability. A new target was also installed. Robustness of the proposed general model was investigated.

### C. on table ‐ inside phantom: transformation

Measurements were performed with the inserts in the phantom and on the table. MLR with dummy variables was used to assess significant differences. Differences of measured HU from *on table* with *inside phantom* were investigated.

### D. dose rate dependence of the IVDT curve

By adjusting the PfN values of the imaging beam, the dose rate can be varied. The IVDT was measured with different dose rates, ranging from 12 MU/min up to 21 MU/min.

### E. Verification of the proposed protocols

First of all, a baseline test was performed to check the difference between kVCT and MVCT dose calculation on head‐and‐neck phantom. A plan was made on a kVCT image and recalculated on an MVCT image using the MVCT IVDT model. The head‐and‐neck phantom^(15)^ incorporates heterogeneities (bone, air). The treatment plan of 2 Gy, using pitch 0.287 and field width 2.5 cm, consists of a cylindrical target volume in the base of the skull. All dose verifications were performed by comparing the 50% volume DVH (ICRU prescription point). The tissue characterization phantom was always scanned immediately before the MVCT scan of a phantom in order to represent the “real” IVDT for comparison with the protocols. Both protocols were tested by using the measurements from before the target change as baseline (determination of c parameter of the response curve). We then applied the proposed transformations and fitting to four new tests in time.

Verification of the first proposed protocol (daily morning scan of two inserts on the table) was done by (a) scanning the two inserts in the morning, (b) applying the proposed transformations and creating the response curve, and (c) recalculating the dose on an MVCT scan of the phantom later that day using the created response curve. This was then compared with a dose calculation using the results of the real IVDT scanned just before the phantom scan. The second protocol was verified the same way, but replacing step (a) by an MVCT scan with the two inserts next to the phantom.

A patient study was performed by comparing the 50% volume DVH of the target volumes. Long‐term performance was evaluated by comparison of dose calculated when using the most extreme IVDT's found throughout the lifetime of two targets. The maximum dose differences are represented of six head‐and‐neck and six pelvic region cases, and one melanoma and one anal canal patient. No breast or lung patients were examined during this study.

## III. RESULTS & DISCUSSION

### A. response curve

#### A.1 Heteroscedasticity of measured HU values

**Figure 1:**
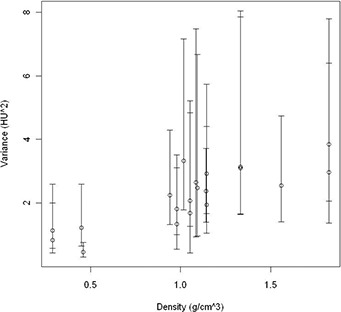
Variance of the measured HU values based on a sequential set of 20 measurements, 95% confidence interval.

The obtained heteroscedasticity is depicted in Fig. 1. In the following parts, this real variance of every insert is used for statistical fitting purposes: weighted least squares fitting (WLS). WLS fitting uses the uncertainty per data point for fitting purposes (e.g., points with high uncertainty get a lower weight for fitting).

#### A.2 General model

**Figure 2:**
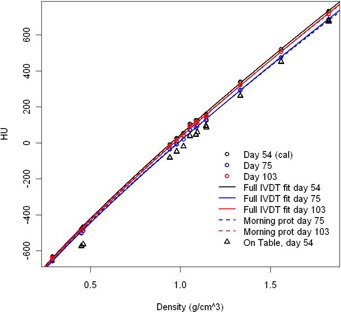
General model fits and the change in time. Results of all inserts are indicated with circles. The corresponding full IVDT fit is represented in full lines. The simultaneously tested morning protocol result is given in dashed lines. Results of a series of measurements of inserts on the table are indicated in triangles. The protocols were calibrated on day 54 (c parameter). Later dates only fit the b parameter to the adjusted *on table* values of the two highest density inserts.

The function $\text{HU}=b\rho^{c}‐1020$ (adjusted $R^{2}=0.9995$, p‐values<0.001) was chosen as it has the most appropriate behavior for (a) low and high densities, (b) values close to vacuum/air, and (c) density $1\;g/{\text{cm}}^{3}$. Uncertainty in the exponent c is the least significant in that region (see Results & Discussion Section B.3). To obtain a correct value for air, the intercept point was taken at ‐1020 (resulting in $0.001\;g/{\text{cm}}^{3}$ density for air) instead of ‐1024 (Note: this could differ between machines, depending on machine calibration.) The values between ‐1024 HU and ‐1000 HU can then be forced to $0\;g/{\text{cm}}^{3}$. The plots are depicted in Fig. 2.

Using this model, the influence of slice thickness was investigated by applying MLR with dummy variables. We see in Table 1 that $H_{0}$ is maintained for the slice thickness. We noticed a small deviation for the Coarse slices compared with both Fine and Normal, but not enough to reject $H_{0}$. We used normal slice thickness for all following measurements.

**Table 1:** Results of comparisons: for various slick thicknesses, before and after airscan, and inserts in phantom and on table. MLR regression with dummy variables.

	*Slice Thickness*			*BAS‐AAS*	*IP‐Table*
	*Fine‐Normal*	*Fine‐Coarse*	*Normal‐Coarse*		
p‐value	0.913	0.262	0.308	<0.001	<0.001

Before airscan=BAS; after airscan=AAS; in phantom =IP, on table = Table.

### B. Stability

#### B.1 Short term: Wald‐Wolfowitz runs test

**Figure 3:**
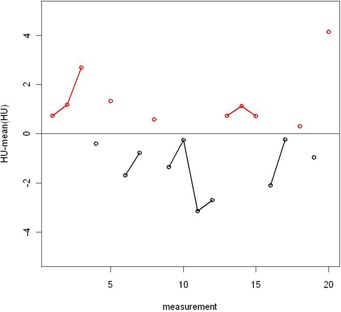
Example of the results of a series of 20 successive measurements for the insert with density $0.98\;g/{\text{cm}}^{3}$. A run corresponds with a series above the mean (red, 6 in this case) or below the mean (black, 5 in this case). This was repeated for all different inserts, over three different series of 20 measurements.

The resulting p‐value is below 0.05 for only two of the 20 inserts (0.02), which indicates random behavior on the short term of 20 successive scans. An example of the results for the insert with density $0.98\;g/{\text{cm}}^{3}$ is depicted in Fig. 3. The other two series on different days (morning and evening) result in a similar behavior. Standard deviations over the whole line are around 2 HU. No extreme values were observed (max deviation 5 HU, normal distribution). This test was performed before and after the magnetron change (instabilities before the change). There were no extreme values or a different behavior observed for our installation. We conclude random behavior on short term with σ=±2 HU (see Fig. 1).

#### B.2 Daily variation, before/after airscan: MLR with dummy variables

The MLR with dummy variables technique was applied for the values in the morning vs. the evening and the same evening before vs. after airscan. A statistically significant difference was observed in the model (see Table 1). However, this shift during the day was only very small (5 HU). A new airscan could compensate most of this very small shift.

This daily shift resulted in only a small change for the dose calculations: 0.2% dose difference for 50% volume of the PTV for several patient cases. For the tested days, no dose rate differences were observed between morning and evening. For dose rates during the day, we refer to Material and Methods Section D.

#### B.3 Long‐term stability and stability after interventions

The IVDT curve was measured and fitted before and after several technical interventions (see Table 2). Both parameters change in time. However, the exponent c ($\text{HU}=b\rho^{c}‐1020$) is (1) of small influence (randomly), (2) of minor influence in the density region of interest, and (3) neither more fluctuating on short term. c can thus be taken as a constant with only minor consequences in bias. Furthermore, around density 1, variations (and thus uncertainties) in c are almost insignificant. When using the mean value of the exponent (c=0.835 in our case), the maximum bias of all different measurements for density $1.823\;g/{\text{cm}}^{3}$ was 7 HU (relative 0.05%). The b parameter results in biases of more than 55 HU (relative 3% over the whole range) and is thus the most important parameter. We also see a significant shift in the parameter b before and after the target change. The change of magnetron resulted in a change in parameter b, but as mentioned before, not in a lower variance or peaks for the HU values in the MVCT images.

**Table 2:** Long‐term parameters of the model. The technical interventions are indicated on the second line (values below the intervention are values obtained after that intervention). b_p_ is the result of the b parameter using the proposed protocols with exponent c as constant from calibration point. The performance of the two protocols (bottom two lines) was tested alongside full measurements of the IVDT curve (b and c). This is also graphically represented in Fig. 2.

*Day*	*1*	*20*	*28*	*36*	*51*	*54*	*55*	*74*	*75*	*90*	*103*
Tech				Mag			Target				
b	1077.6	1059.9	1066.9	1086.1	1064.1	1064.5	1025.7	1037.4	1029.5	1032	1050.3
c	0.840	0.837	0.835	0.839	0.834	0.831	0.843	0.835	0.843	0.840	0.841
$b_{p1}$	Daily prot.					cal			1031.9	1035.3	1054.3
$b_{p2}$	Pat Prot.					cal		1033.7	1034.9	1035.0	1062

This leads to the fit of a single parameter b in order to obtain the IVDT curve. A QC procedure (for example, biannual) could be applied to check the constance of the “c” parameter.

### C. Inside phantom ‐ on table: transformation

Measurements were performed with the inserts in the phantom and on the table (six sets of measurements in time). There is a significant difference in model for the different setup (see Table 1 and Fig. 2). We investigated the transformation of measured HU from *on table* to *inside phantom*. The difference in HU between *on table* and *inside phantom* is close to an individual constant for each one of the highest density inserts. However, the HU difference between *on table* and *in phantom* is not constant over the whole density/insert range: each insert has a different factor Table‐InPhantom (most likely due to scattering contributions) and shows higher variability for the lowest density inserts. We decided to use the $1.56\;g/{\text{cm}}^{3}$ and $1.823\;g/{\text{cm}}^{3}$ insert with δHU(in‐table) of respectively 69 (± 5) HU and 53 (± 8) HU (for our installation) as these show the lowest δHU values. One could use a calibrated phantom with regular density, but this phantom should have a larger size than the inserts as the variability for the lower/medium density inserts *on table* is a lot higher than the high‐density inserts.

If the inserts are next to the patient, an additional bias can be introduced due to the presence of the patient next to the inserts. We evaluated this by comparing the values of the inserts next to the head phantom with the values of the inserts on the table. We did not incorporate this bias of up to 10 HU in the model as this can vary from patient to patient. If the inserts are above the patient (nothing next to the inserts), this bias is removed.

### D. Dose rate variation of the IVDT curve

**Figure 4:**
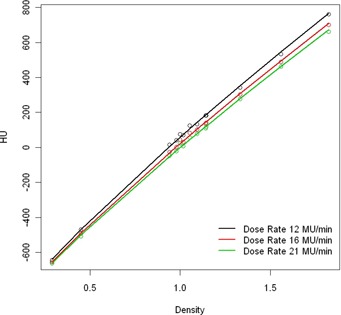
Example of dose rate dependence. The PfN values were adjusted in order to change the dose rate. The b parameter of the model $\text{HU}=b\rho^{c}‐1020$ follows the relation depicted in Fig. 5.

**Figure 5:**
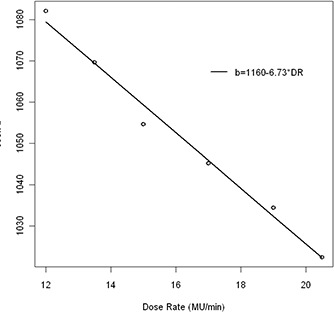
Relation between the parameter b of the model $\text{HU}=b\rho^{c}‐1020$ and the dose rate. If the dose rate during the day changes, the b parameter can be adjusted.

The results of the measurements are depicted in Figs. 4 and 5. We see that the proposed model follows perfectly the dose rate: the slope parameter b is a linear function of the dose rate (Fig. 5). If the dose rate varies during the day, the IVDT curve can be adjusted using these results.

### E. Verification of the protocol

**Figure 6:**
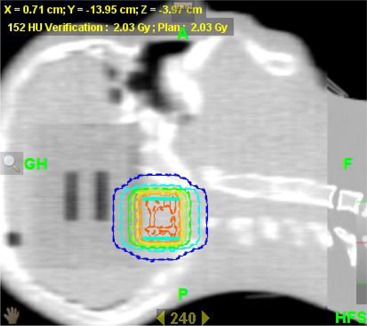
Fused MV/kV image of the head and neck phantom. kV calculated isodoses (solid lines) and MV calculated isodoses (dashed) are represented. A 0.2% dose difference for the 50% PTV was obtained for calculations on the MVCT images (using correct IVDT), compared with the kVCT images.

The baseline test (dose calculation on kVCT vs. MVCT) using the response function on the head‐and‐neck phantom resulted in a 0.2% dose difference (see Fig. 6).

If we now combine the transformation Table‐InPhantom with the fitting results from last paragraph, we can obtain the full IVDT curve by fitting the b parameter to the transformed value of the two highest density inserts on the treatment table. The HU value of an insert next to the patient depends, however, on the size of patient. Indirectly, the resulting HU value can then differ by 10 HU for head‐and‐neck cases. The difference will be larger for pelvis cases, but due to the limited FOV, this was not evaluated. This results in dose differences of 0.3%.

We verified the daily protocol (p1) of scanning two inserts on the table in the morning by using the model and transformation from just before the target change (day 51). We applied this protocol at days 75, 90, and 103 and evaluated the protocol with full IVDT measurements of these days (Table 2 (p1)). We also verified the patient specific protocol (p2) on days 74, 75, 90, and 103 the same way, but with two inserts next to the head‐and‐neck phantom (phantom, in order to avoid patient specific geometrical variations). Finally we recalculated the dose on the phantom using the reconstructed IVDT. We noticed a drop of dose differences to below 0.4% dose.

The final summary of the resulting dose differences due to the IVDT instability is represented in Table 3. These were evaluated for several patient cases, by applying the correct and most extreme IVDTs and calculating the dose for the 50% PTV DVH. When applying the protocols the dose deviations are significantly reduced from 3% to 0.4%.

**Table 3:** Maximum observed dose deviations for the 50% DVH of 6 pelvic and 6 H&N cases.

	*Pelvis*	*H&N*
kVCT ‐ MVCT (phantom)		0.2%
Short Term (successive scans)	<0.1%	<0.1%
Morning/evening (no DR change)	0.2%	0.2%
Long Term (no tech intervention)	1.6%	0.7%
New Target	1.5%	0.7%
Whole Period	3.2%	1.4%
Application Model	0.4%	0.3%

## IV. CONCLUSIONS

We propose (1) a method to use a response function instead of HU values, and (2) two protocols to ameliorate dose calculations on tomotherapy MVCT images — a daily (recommended) and a patient specific protocol to lower the dose differences from 3% to 0.4%. This can result in less type I or II errors when deciding to replan a patient. No erratic behavior was observed on short term or within a day, only a slow drift during the day. The daily and long‐term changes were most important. The reason of the nonlinearity could be due to scatter contribution, but requires further investigation.

The daily protocol proposes a morning airscan‐like procedure with only two inserts on the table for correction of the base function. Dose rate variations during the day are corrected for with the dose rate‐IVDT relation. The patient specific protocol proposes the addition of two inserts on the table next to the patient (or further on the table). By measuring the HU values of these inserts and applying the proposed transformations and fit, we can reconstruct the IVDT curve of that moment. The whole phantom could be used also, but reducing the measurement to two inserts simplifies the procedure in the morning, at only a slight uncertainty increase.

We proposed the use of two inserts with highest densities. In theory, only a single insert is required to create the IVDT of that moment. However, the highest density inserts are more optimal: (a) they show lower variability *on table*, and (b) their values are higher, thus result in lower uncertainty on the curve. Any other (calibrated) density insert could also be used, but when used *on table*, it should be of larger size than the current inserts.

The morning procedure is the most practical, but results in variations throughout the day (0.4% dose for head and neck). The patient‐specific procedure is a snapshot of that moment, but it introduces a possible patient‐specific bias if the inserts are next to the patient (0.3% dose). Also, this patient‐specific procedure is not always feasible due to the limited field of view (pelvis cases). The inserts can only be placed above the patient in the gantry for head‐and‐neck patients and this will take longer scan time.

In conclusion, we propose to implement the response function along with the simplified morning procedure with two inserts on the table (or large calibrated single medium density phantom). Dose variations throughout the day can be corrected for using the proposed dose rate model. In the current version of TomoTherapy, several “b” value curves could be created. The “b” value can be recalculated for each patient, and thereafter the IVDT curve of that moment can be picked from the list of corresponding “b” values in the TPS.
